# Clinical Success Rates of Dental Implants with Bone Grafting in a Large-Scale National Dataset

**DOI:** 10.3390/jfb17010046

**Published:** 2026-01-15

**Authors:** Mordechai Findler, Haim Doron, Jonathan Mann, Tali Chackartchi, Guy Tobias

**Affiliations:** 1 Oral Medicine Unit, Sheba Medical Centre Tel-Hashomer, Ramat Gan 526210, Israel; findlermo@gmail.com; 2 Research Unit Maccabi-Dent, Tel Aviv 680129, Israel; haim_d@maccabi-dent.com; 3 Department of Community Dentistry, School of Dental Medicine, Hebrew University and Hadassah, Jerusalem 91120, Israel; mannhadassah@gmail.com; 4 Department of Periodontology, School of Dental Medicine, Hebrew University and Hadassah, Jerusalem 91120, Israel; tali222@hotmail.com

**Keywords:** dental implants, bone augmentation, implant survival, socioeconomic status, retrospective study

## Abstract

Objective: To evaluate the clinical success outcomes and risk factors associated with dental implants placed with simultaneous bone augmentation in a large-scale, real-world cohort. Methods: A retrospective analysis was conducted on 158,824 implants, including 45,715 Dental Bone Grafts, placed between 2014 and 2022 within a national healthcare network. Multivariate Generalized Estimating Equations were utilized to assess the impact of demographic, anatomical, and procedural variables on implant failure. Results: The augmented cohort demonstrated a high clinical success rate of 97.83% (2.17% failure), statistically comparable to the general implant population. Failures were predominantly early (<1 year), accounting for 70% of losses. Significant independent risk factors included immediate implant placement (3.08% failure vs. 2.07% for delayed), male gender, and maxillary location. Notably, low socioeconomic status (SES) emerged as a significant predictor, with a failure rate of 3.07% compared to 2.06% in high-SES groups. Conclusions: Simultaneous bone augmentation is a predictable modality that does not inherently increase implant failure risk, supporting the stabilization hypothesis. However, failure is modulated by specific variables. The identification of lower SES, male gender, and immediate placement as significant risk indicators highlights the necessity for personalized risk assessment and targeted protocols to optimize outcomes in augmented sites.

## 1. Introduction

### 1.1. Dental Implants and the Need for Adequate Bone Support

Dental implants are widely established as a predictable and durable therapeutic modality for the rehabilitation of edentulous spaces, with long-term clinical success rates frequently exceeding 95% under optimal conditions [1,2]. However, clinical success is intrinsically linked to the availability of adequate alveolar bone volume to ensure primary stability and long-term biological maintenance [3]. Tooth loss almost invariably precipitates progressive alveolar ridge resorption, a sequela driven by trauma, periodontal pathology, infection, or the lack of mechanical stimulation associated with prolonged edentulism [4]. Consequently, a substantial proportion of implant sites present with compromised osseous dimensions at the treatment planning stage, constituting a significant clinical challenge [5,6].

To mitigate these anatomical deficiencies, a spectrum of bone augmentation and regenerative protocols has become integral to contemporary implant dentistry [7]. Standard interventions include socket preservation post-extraction, guided bone regeneration (GBR) utilizing particulate grafts and barrier membranes, and sinus floor elevation in the atrophic posterior maxilla [8,9]. Over the last decade, scientific inquiry into these procedures has intensified, with augmentation-related research now comprising a significant fraction of the implant literature [10]. This trend underscores the prevalence of osseous defects in routine practice and the pivotal role of grafting in broadening the indications for implant therapy [11].

### 1.2. Timing of Implant Placement: Concepts and Clinical Implications

A critical determinant in treatment planning is the timing of implant placement relative to tooth extraction. According to consensus classifications, such as those proposed by the International Team for Implantology (ITI), placement protocols are categorized as immediate (Type 1), early (Types 2 and 3), or delayed in fully healed sites (Type 4) [12,13].

Immediate placement offers distinct benefits, including abbreviated treatment timelines and the potential preservation of soft tissue architecture [14]. However, this approach is often complicated by the presence of peri-implant gaps, which necessitate simultaneous bone grafting [15]. While randomized clinical trials and meta-analyses generally report high clinical success rates across all timing protocols, immediate placement is frequently regarded as technique-sensitive [16,17].

Divergent clinical perspectives persist regarding the optimal management of the extraction socket [18]. Concerns regarding residual infection, compromised primary stability, and unpredictable healing dynamics have led to an ongoing debate [19]. While some evidence suggests that immediate placement combined with grafting yields outcomes superior to delayed protocols without grafting, other studies report no significant advantage [20]. This underscores the lack of consensus regarding the ideal synergy between placement timing and augmentation to minimize marginal bone remodeling.

### 1.3. Implant Clinical Success Rates in Augmented Bone: Evidence and Controversies

The comparative clinical success of implants placed in augmented versus pristine native bone remains a subject of extensive investigation. Early hypotheses postulated that graft remodeling, delayed vascularization, or inferior bone density might impede osseointegration [21].

Subsequent research has largely refuted these concerns. Large-scale retrospective analyses and systematic reviews consistently demonstrate that implants in well-executed augmented sites—including complex sinus elevations—achieve clinical success rates statistically indistinguishable from those in native bone [22,23,24]. Nevertheless, controversy persists regarding the impact of specific graft materials. While autogenous bone and xenografts are associated with predictable outcomes, certain synthetic substitutes have been linked to marginally reduced implant failure rates in specific cohorts [25,26]. These findings suggest that while augmentation is not inherently detrimental, prognosis may be modulated by procedural nuances and material selection.

### 1.4. Early Implant Failure as a Distinct Clinical Outcome

Early implant failure—defined as failure occurring prior to or shortly after prosthetic loading—constitutes a critical clinical endpoint, typically indicative of a failure to achieve osseointegration [27]. Early failures are fundamentally multifactorial and heavily influenced by patient-specific variables, including smoking habits, pharmacological history, and systemic health [28,29]. From a biological standpoint, the impact of bone grafting on early failure is the subject of conflicting hypotheses. The stabilization hypothesis suggests that augmentation reduces risk by restoring local anatomy and enhancing mechanical anchorage [24]. Conversely, the complexity hypothesis argues that implants placed in native bone, particularly if integration is incomplete or remodeling is active, may alter healing cascades and increase susceptibility to failure compared to native bone [30]. The net effect of bone grafting on early failure risk thus remains unresolved in the current literature.

### 1.5. Socioeconomic Factors and Emerging Risk Dimensions

Beyond biological and technical parameters, socioeconomic status (SES) represents a significant yet under-investigated determinant of implant outcomes [31]. Patients with lower SES may encounter barriers such as restricted access to maintenance care, lower health literacy, and a higher prevalence of behavioral risk factors [32]. Preliminary data suggest that disparities in insurance coverage and continuity of care may translate into differential clinical outcomes, yet robust, large-scale evidence linking SES to implant failure remains scarce [33,34].

### 1.6. Study Rationale, Hypotheses and Aim

Given the multifactorial etiology of implant failure and the low incidence of early loss, large retrospective datasets are essential for detecting subtle risk patterns [35]. The present study leverages the comprehensive digital records of a large healthcare organization to evaluate dental implants placed with Dental Bone Grafting Implants (DBGIs).

We hypothesize that: (1) implants placed with bone grafting do not exhibit higher overall or early failure rates compared to the general implant population; and (2) failure risk in DBGIs is significantly modulated by implant timing, anatomical location, prosthetic rehabilitation mode, and patient demographic factors, specifically socioeconomic status.

The primary aim of this study is to assess implant clinical success and failure patterns in augmented sites within a real-world clinical setting.

## 2. Materials and Methods

### 2.1. Study Design and Data Source

This study was conducted as a retrospective cohort analysis using electronic dental records from Maccabi Dent, a nationwide dental service operating within Maccabi Health Services, Israel. The database systematically records all dental procedures using standardized codes, allowing longitudinal tracking of treatments and outcomes across affiliated clinics. All dental implant placements performed between 1 January 2014 and 31 December 2022 were extracted, along with associated bone grafting and prosthetic procedure records.

The study adhered to the Declaration of Helsinki and was approved by the Institutional Review Boards of Maccabi Health Services (Protocol MHS-0090-20) and Assuta Medical Center (Protocol ASMC-0032-20). As the analysis was based on de-identified registry data, informed consent was waived.

### 2.2. Cohort Definition and Variables

From a total of 158,824 implants, we identified implants placed concurrently with bone augmentation procedures. These were defined as DBGIs, operationally identified by the presence of a bone graft procedure code recorded on the same day and anatomical site as implant placement. Augmentation techniques included socket grafting, guided bone regeneration, ridge expansion with grafting, block grafts, and sinus floor elevation. Implants placed without same-day grafting served as a comparison group. In addition, sites with a history of prior staged augmentation (e.g., ridge preservation or earlier grafting) were identified separately.

For each implant, we extracted patient age and sex, implant placement date, jaw region, number of implants per patient, implant timing (immediate vs. early/delayed placement), prosthetic restoration type (fixed vs. removable), and clinic location. Immediate placement was defined as implant placement recorded on the same date and site as tooth extraction; all others were classified as early/delayed placements. Clinic location served as a proxy for socioeconomic status (SES) based on an internal index that categorized clinics into low, medium, or high SES groups. This classification aligns with the Israel Central Bureau of Statistics (CBS) methodology, which assigns a socioeconomic rank to residential areas via a clustering system. Accordingly, the study population was divided into three primary cohorts: clusters 1–3 representing low SES, clusters 4–6 representing medium SES, and clusters 7–9 representing high SES.

### 2.3. Outcome Definition

Implant failure was defined as removal of the implant or documentation of failure in the database. Failures were further categorized as early (≤1 year post-placement) or late (>1 year). Implants without a failure record were considered surviving and censored at the study end date. Failures were also classified as occurring before or after prosthetic restoration when applicable.

### 2.4. Data Cleaning

Data underwent systematic validation to ensure logical consistency. Erroneous records (e.g., restorations dated before implant placement), duplicate entries, and canceled procedures were excluded. When implant placement and removal were recorded on the same day, the case was treated as an ultra-early failure unless contradictory information existed. Each implant was ultimately represented by a single consolidated record. Approximately 22,600 implants lacked restoration records by study end; these were retained for failure analyses but excluded from restoration-dependent analyses.

### 2.5. Statistical Analysis

All analyses were performed using IBM SPSS Statistics (Version 27.0; IBM Corp., Armonk, NY, USA). Descriptive statistics summarized continuous variables (mean, standard deviation) and categorical variables (frequencies, percentages). Implant clinical success was treated as a binary outcome (failure vs. success).

Univariate associations between implant failure and categorical predictors were assessed using chi-square tests (α = 0.05). To account for clustering of multiple implants within patients and potential confounding, multivariable analysis was conducted using Generalized Estimating Equations (GEE) with a binary logistic link, exchangeable correlation structure, and robust standard errors. Variables entered into the multivariable model included sex, SES, implant timing, jaw region (six anatomical zones), restoration type, and concurrent bone grafting. Results are presented as odds ratios (ORs) with 95% confidence intervals. A subgroup GEE analysis evaluated early failure risk in implants placed in previously augmented sites.

No imputation was performed for missing data; cases with missing key variables were excluded listwise. Given the large sample size, both statistical significance and clinical relevance were considered when interpreting results.

## 3. Results

### 3.1. Cohort Characteristics and Implant Placement Details

After exclusion of records with data inconsistencies (e.g., duplicate entries) and completion of a structured data refinement process, the final dataset comprised 158,824 dental implants placed in 53,874 patients. The cohort included 29,501 females, 24,177 males, and 196 patients with no definitive gender classification. The mean patient age at the time of implant placement was 55.54 ± 11.93 years (range 18–88).

Of all implants, 133,892 ultimately received fixed prosthetic restorations, while 3513 were restored with removable dentures. An additional 22,607 implants had no recorded restoration by the end of follow-up, likely reflecting recent placement near the study endpoint, early failure prior to reconstruction, or restoration performed outside the reporting framework.

A total of 45,715 implants were placed with concomitant bone augmentation procedures in 17,600 patients. Within this augmented implant group, 11.4% (*n* = 4682) were placed immediately at the time of tooth extraction, whereas 88.6% (*n* = 41,033) were placed after a healing period (early or delayed placement). Immediate augmented implants demonstrated a significantly higher failure rate compared with early or delayed augmented implants (3.08% vs. 2.07%, *p* < 0.001). This association remained significant in the multivariable GEE analysis (odds ratio 1.487, *p* < 0.000).

**Figure 1:**
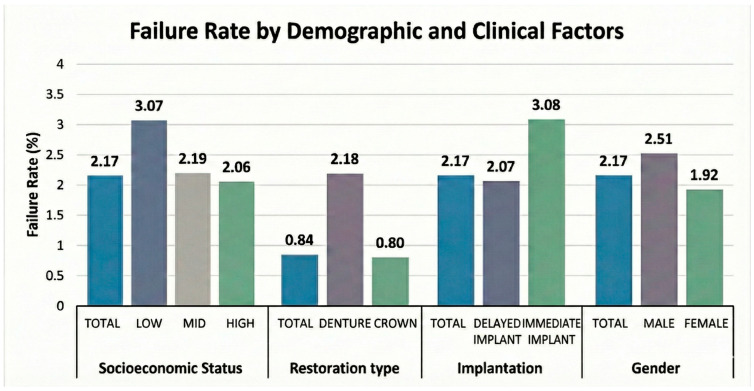
Failure Rates Stratified by Demographic and Clinical Variables.

More females than males underwent augmented implant placement (10,186 vs. 7414 patients, respectively). Despite this, implant failure occurred significantly more frequently in males (*p* = 0.0002). The distribution of failures according to sex, implant timing, restoration type, and socioeconomic status is summarized in Figure 1.

### 3.2. Implant Clinical Success and Timing of Failure

Within the augmented implant cohort, 994 implants failed during the observation period. Of these failures, 696 (70%) occurred within the first year following placement and were classified as early failures indicative of impaired osseointegration. The remaining 298 failures (30%) occurred more than one year after placement. A total of 324 failures (32.6%) occurred after prosthetic reconstruction, while the remainder occurred prior to restoration.

**Figure 2:**
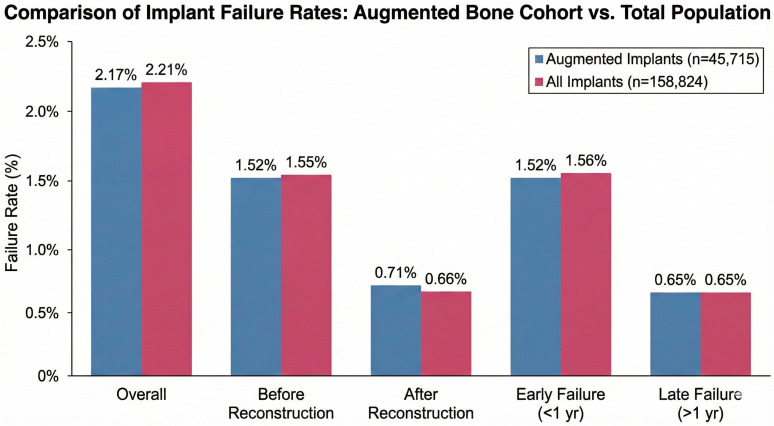
Comparative Analysis of Failure Rates in Augmented versus Total Implant Populations.

Across the entire dataset of 158,824 implants, 3507 failures were recorded. Of these, 2476 (70%) occurred within the first year and 1031 (30%) after more than one year. Similarly, 2455 failures (70%) occurred before reconstruction and 1052 (30%) after reconstruction. between augmented implants and the overall implant population. These comparisons are presented in Figure 2 and Table 1.

The mean interval from implant placement to failure was 13.77 ± 17.31 months, reflecting a concentration of failures early after surgery with a smaller proportion occurring later. Prosthetic restorations were delivered after a mean of 6.19 ± 3.57 months. Among implants that failed after restoration, the mean time from loading to failure was 22.9 ± 21.81 months.

### 3.3. Implant Location and Failure Distribution

Implant failure rates differed significantly by anatomical location, *p* < 0.01. Failure was consistently more frequent in the maxilla than in the mandible. The highest failure rates were observed in the maxillary molar region (3.36%) and the maxillary incisor region (3.09%), both of which were significantly higher than corresponding mandibular regions (GEE *p* < 0.0004 and *p* < 0.000, respectively). The maxillary premolar region also exhibited a higher failure rate than the mandibular premolar region (2.16% vs. 1.16%, *p* < 0.0056).

In contrast, failure rates in mandibular regions were lower and relatively uniform, ranging from 1.61% to 1.69% across incisor, premolar, and molar areas.

**Figure 3:**
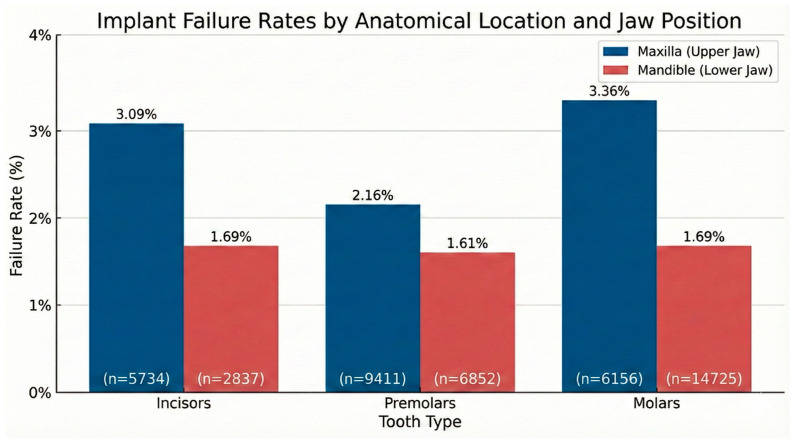
Implant failure rates stratified by anatomical site and jaw position (maxilla vs. mandible).

The regions most frequently requiring bone augmentation were the mandibular molar region (32.2%) and the maxillary premolar region (20.6%), reflecting common clinical indications for grafting in areas with limited bone volume or challenging anatomical conditions, Figure 3.

GEE with weights analysis.

### 3.4. Prosthetic Restoration Type and Failure

**Figure 4:**
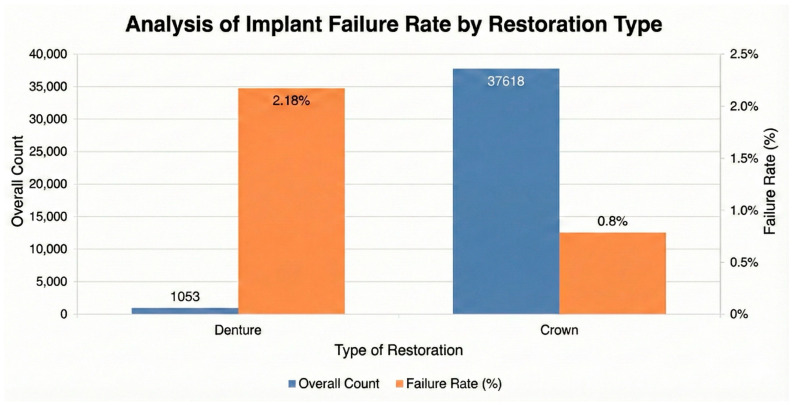
Comparison of overall implant count and failure rate stratified by type of prosthetic restoration (denture vs. crown).

Among the 324 failures that occurred after prosthetic rehabilitation, 301 implants supported fixed restorations (crowns), while 23 implants supported removable prostheses. When failure rates were evaluated relative to the number of restored implants, those supporting removable dentures exhibited a higher proportion of post-loading failures compared with implants supporting fixed restorations, as illustrated in Figure 4.

## 4. Discussion

The primary aim of this large-scale retrospective analysis was to evaluate the clinical outcomes of dental implants placed in conjunction with DBGIs within a national dental care network. Our results, derived from a cohort of over 158,000 implants, provide robust evidence that implants placed in augmented sites achieve high clinical success rates comparable to those placed in native bone. The overall failure rate of approximately 2.2% observed in our study aligns with, and in some cases is superior to, reports from recent systematic reviews and longitudinal studies [36,37]. Specifically, the 97.8% success rate for augmented implants supports the “stabilization hypothesis,” suggesting that contemporary augmentation techniques effectively restore anatomical integrity, thereby mitigating the risks associated with compromised bone volume [38]. A critical finding in our study was the temporal distribution of failures. Across both augmented and non-augmented cohorts, approximately 70% of failures occurred within the first year following placement. This clustering of “early failures” indicates that the predominant challenge remains the establishment of osseointegration rather than late-stage biological complications such as peri-implantitis [39]. Importantly, augmented sites did not exhibit a higher propensity for early failure compared to native sites. This finding counters the “complexity hypothesis,” which posits that the introduction of graft materials might inherently disturb initial healing dynamics. Instead, our data suggests that once the critical window of osseointegration is passed, augmented bone functions as a stable long-term foundation, with late failure rates dropping to ~0.65% annually [40]. Our multivariate analysis identified patient gender as a significant modulator of implant failure rates. Male patients exhibited a failure rate of 2.5% compared to 1.9% in females, representing a statistically significant independent risk factor. While some historical literature has reported equivocal findings regarding gender [41], our results corroborate large-scale studies indicating a male predisposition to implant failure [42]. This disparity is likely multifactorial, potentially attributable to behavioral differences such as higher rates of tobacco use, poorer oral hygiene compliance, and a higher prevalence of parafunctional habits like bruxism among males [43]. Biological variations in bone density and hormonal profiles may also play a role, although behavioral and environmental factors are generally considered more impactful in the context of implant therapy [44].

Perhaps the most novel finding of this investigation is the significant association between SES and implant failure.

Our data demonstrates a clear clinical gradient: patients in the lowest SES quintile experienced a failure rate of 3.07%, a statistically significant increase compared to the 2.06% observed in the highest SES group. This disparity persisted even after rigorous adjustment for clinical variables such as bone density, implant location, and systemic health markers, suggesting that social determinants of health exert a measurable, independent influence on long-term osseointegration.

While historical data has established a link between low SES and the severity of periodontal disease [45], recent evidence suggests that the impact on implant outcomes is driven by more nuanced factors than mere access to care.

The mechanisms likely involve a convergence of three primary domains:

1. Behavioral and Lifestyle Clusters: Lower SES is frequently associated with higher rates of tobacco use, suboptimal nutritional intake (specifically Vitamin D and antioxidants essential for bone healing), and poorer glycemic control, all of which are known inhibitors of implant success [46].
2. Psychosocial Stress and Biology: Chronic “weathering” or allostatic load—the physiological wear and tear resulting from chronic socioeconomic stress—may impair the immune response and osteoblast activity, potentially compromising the early healing phase [47].
3. Health Literacy and Maintenance: Disparities in health literacy often translate to lower adherence to complex post-operative protocols and professional maintenance schedules [48]. In lower SES populations, “reactive” dental attendance often replaces “preventative” maintenance, allowing peri-implant mucositis to progress to irreversible peri-implantitis undetected.

In the context of a universal healthcare setting, where surgical access is ostensibly equitable, these findings expose a paradox: equal access to the procedure does not guarantee equal outcomes. The data suggests that clinical success is heavily contingent upon the patient’s post-operative environment [49]. Consequently, achieving equity in implant dentistry requires a shift from a “one-size-fits-all” approach to a model of proportionate universalism, where vulnerable populations receive intensified post-operative support, culturally tailored education, and subsidized maintenance pathways [50].

Regarding surgical protocols, our data revealed that immediate implant placement is a significant risk indicator. Immediate implants in augmented sites failed at a rate of 3.08%, compared to 2.07% for delayed protocols. This aligns with the consensus that immediate placement is a technique-sensitive procedure where achieving primary stability and managing the socket gap present unique challenges [51]. The higher incidence of failure in immediate cases, particularly in the pre-restorative phase, likely reflects difficulties in achieving adequate initial stability or subclinical infection at the extraction site [49]. Conversely, the finding that bone augmentation per se was not a major risk factor-once timing was accounted for-reinforces the safety of GBR procedures. While the multivariate model showed a marginal increase in failure odds for grafted sites (OR ~1.1), this is likely a reflection of selection bias, where grafting is utilized in sites with more severe anatomical deficiencies [52].

Anatomical location proved to be a strong predictor of outcomes, reaffirming the maxillary posterior region as a high-risk zone. Maxillary implants failed at nearly double the rate of mandibular implants, a discrepancy widely attributed to differences in bone density (Type III and IV bone) [53]. The anterior maxilla also demonstrated elevated failure rates, which may be confounded by the high frequency of immediate placement and high esthetic demands in this zone, potentially leading to failures where implants are removed due to malposition rather than biological loss [54]. In contrast, the anterior mandible showed the highest success rates, consistent with its dense trabecular bone quality [55]. However, an interesting nuance was the elevated risk when the anterior mandible required augmentation, suggesting that when this typically robust site is compromised (e.g., by large defects), the risk profile shifts significantly.

Prosthetic factors also influenced implant failure rates, with removable overdentures associated with higher failure rates (2.18%) compared to fixed crowns (0.80%). This supports literature suggesting that the biomechanical loading patterns of overdentures, combined with the often reduced number of supporting implants, create a higher risk environment [56]. Furthermore, the overdenture cohort likely comprises an older demographic with more systemic comorbidities, although age itself was not a primary driver in our model. This finding emphasizes the need for meticulous biomechanical planning and maintenance for removable prostheses [57].

### Limitations

This study’s retrospective design, relying on administrative coding, presents several limitations. While the large sample size provides substantial statistical power, we lacked granular data on key confounding variables such as smoking status, history of periodontitis, and the specific graft materials utilized. Furthermore, our cohort included a variety of surgical approaches, such as sinus elevation and horizontal augmentation. As these procedures may have inherently different failure rates, the current lack of granular data prevented us from performing a dedicated subgroup analysis on simultaneous horizontal augmentation. This procedural heterogeneity may introduce bias, as mixing distinct clinical techniques could mask specific outcomes associated with each approach. Additionally, our definition of failure was binary (implant removal); consequently, we could not account for implants experiencing marginal bone loss that remained in situ, which may lead to an underestimation of the true prevalence of peri-implantitis. Finally, the follow-up period was variable, and while the GEE model accounts for intra-subject correlation, a comprehensive long-term implant failure rates (e.g., a 10-year Kaplan–Meier curve) was not feasible for the entire cohort.

## 5. Conclusions

In conclusion, this large-scale analysis confirms that dental implants placed with simultaneous bone augmentation achieve high clinical success rates indistinguishable from those in native bone in a general population. The identification of male gender, low SES, and immediate placement as specific risk factors suggests that while the surgical technique of augmentation is predictable, patient and protocol selection remain critical. Future research should focus on prospective evaluations of specific graft materials and deeper investigations into the biological and behavioral mechanisms driving the SES and gender disparities observed. Personalized risk assessment models incorporating these demographic and clinical variables could further optimize treatment planning and patient outcomes in implant dentistry.

## Figures and Tables

**Table 1:** GEE analysis with weights.

Risk for Failure According to Subset (with Weights)	Odds Ratio	5%	95%	*p* Value	Weights Ratio	Effect
Incisors	0.536	0.379	0.758	0.0004	2.02	Higher failure rate for upper incisors compared to lower
Premolar	0.709	0.556	0.904	0.0056	1.37	Higher failure rate for upper premolar compared to lower
Molar	0.492	0.405	0.598	0	0.42	Higher failure rate for upper molar compared to lower

## Data Availability

The original contributions presented in this study are included in the article. Further inquiries can be directed to the corresponding author.

## Abbreviations

GBR	Guided Bone Regeneration
ITI	International Team for Implantology
SES	Socio Economic Status
DBGI	Dental Bone Grafting Implants
GEE	Generalized Estimating Equations
OR	Odds Ratio
